# Identifying genetic variation affecting a complex trait in simulated data: a comparison of meta-analysis with pooled data analysis

**DOI:** 10.1186/1471-2156-6-S1-S97

**Published:** 2005-12-30

**Authors:** Xiaodong Wu, Donghui Kan, Richard S Cooper, Xiaofeng Zhu

**Affiliations:** 1 Department of Preventive Medicine and Epidemiology, Loyola University Chicago Medical Center, Maywood, IL, USA

## Abstract

We explored the power and consistency to detect linkage and association with meta-analysis and pooled data analysis using Genetic Analysis Workshop 14 simulated data. The first 10 replicates from Aipotu population were used. Significant linkage and association was found at all 4 regions containing the major loci for Kofendrerd Personality Disorder (KPD) using both combined analyses although no significant linkage and association was found at all these regions in a single replicate. The linkage results from both analyses are consistent in terms of the significance level of linkage test and the estimate of locus location. After correction for multiple-testing, significant associations were detected for the same 8 single-nucleotide polymorphisms (SNP) in both analyses. There were another 2 SNPs for which significant associations with KPD were found only by pooled data analysis. Our study showed that, under homogeneous condition, the results from meta-analysis and pooled data analysis are similar in both linkage and association studies and the loss of power is limited using meta-analysis. Thus, meta-analysis can provide an overall evaluation of linkage and association when the original raw data is not available for combining.

## Background

Identifying the susceptibility genes for human complex traits, such as obesity, diabetes, and hypertension, represents a challenging task for human geneticists. Due to the moderate effect of each gene on the trait, it is difficult to acquire enough power to detect all disease susceptibility genes in one study with a moderate sample size. One potential solution to this challenge is to combine the primary studies to increase the power to identify the genes with small effects. When the raw data is available, pooling raw data should be the most powerful method to combine studies. However, in practice, raw data is usually hard to obtain and the alternative is to pool the results (meta-analysis) of the primary studies instead.

Although meta-analysis has been widely used in clinical trials and epidemiological studies, it is a relatively new approach in linkage studies. Methods for meta-analysis of linkage analysis can be roughly divided into two categories. In the first category, individual effect size and its variance are available for each study and can be combined using a fixed-effects or random-effects model to obtain an overall effect. Li and Rao [1] proposed to use regression-coefficient estimates from the Haseman-Elston (HE) regression [2] as effect size and combine them using a random-effects model in sib-pair analysis. Gu et al. [3] developed a similar approach. They used the proportion of alleles shared identical by descent (IBD) among selected sib pairs as their effect size. The advantage of using such methods is that heterogeneity among studies can be estimated and tested, and an overall effect size then estimated. The second category includes methods for combining significance levels, such as *p*-values or LOD scores. The advantage of such methods is that it does not require a common effect size, only that every study tests the same null hypothesis. Thus, these methods are quite general and flexible to use. The very first method for combining results from different studies to obtain consensus was developed by R. A. Fisher in 1925 [4]. Fisher's method of combining *p*-values is remarkably general and easy to use. Wise et al. [5] developed a nonparametric meta-analysis method for genome scans (GSMA) that is based on either *p*-value or LOD score ranks. In this method, each chromosome is separated into independent bins with equal spacing, and the linkage evidence for each bin is ranked in each study. To combine the linkage evidence from different studies, an average rank is calculated for each bin across the studies.

The main aim for this analysis is to identify the underlying genetic factors responsible for Kofendrerd Personality Disorder (KPD) using pooled data analysis and meta-analysis and to further compare the power to detect linkage and association with these two methods.

## Methods

### Data selection

The analysis was performed in the Aipotu population. The diagnosis for KPD in this population is based on all 3 different criteria, which is the most heterogeneous compared with the other 3 populations and represents the most realistic case. Replicates 1–10 were selected for the analysis, which represents a reasonable sample size for the meta-analysis of genome scans in reality.

### Combined linkage analysis

Fisher's method of combining *p*-values was used in our analysis considering that 1) in practice, the designs and applied statistical methods are usually different in linkage analyses and it is difficult to obtain a common effect size for combining; 2) we can combine significance level at every marker location using Fisher's method, but only at every bin (~30 cM) using GSMA.

Linkage analysis with microsatellite markers was performed using a nonparametric allele-sharing method [6] implemented in software ALLEGRO [7] in each of the 10 replicates and also in the pooled data. This method evaluates linkage by testing excessive IBD sharing within affected relatives. We chose *Z*_*lr *_[6] as the test statistic for linkage. Under the null hypothesis of no linkage, *Z*_*lr *_asymptotically follows a normal distribution. We then combined the results of the 10 primary genome scans using Fisher's method of combining *p*-values [4]. This method is based on the observation that if *n *independent tests are made of the same hypothesis, then we can calculate a combined *p*-value for all *n *tests by , where *p*_*i *_is the significance level for study *i*. The combined *p*-value is asymptotically distributed as a chi-square distribution with *2xn *degrees of freedom. Under the null hypothesis of no linkage, 4.6 × LOD follows a chi-square distribution with 1 degree of freedom. In practice, the test is one-sided because it is only declared significant when  < 1/2, where  is the estimated recombination rate. The combined *p*-value was transformed to LOD scores by calculating the quantile of the chi-square distribution.

### Combined association analysis

Hardy-Weinberg equilibrium (HWE) was tested in founders using software package ARLEQUIN [8]. Pair-wise linkage disequilibrium (LD) was calculated as D' and r^2 ^[9] using ldmax routine in software GOLD  in each replicate as well as in pooled data. The *p*-values were corrected for multiple testing for the number of single-nucleotide polymorphisms (SNPs) tested for association. To account for the non-independence between these SNPs, a spectral decomposition method was used to obtain the effective number of independent SNPs [10]. This method has been implemented in the software SNPSpD . SNPs with allele frequency less than 5% were excluded from the analysis.

To further narrow down the regions containing the genetic variants for KPD, we applied family association test implemented in software package FBAT (version 1.5.1) [11] for all of the SNPs within the 1-LOD intervals of linkage peaks from fine mapping study. FBAT builds on the original TDT method [12] and in particular puts tests of different genetic models, tests of different sampling designs, tests involving different disease phenotypes, tests with missing parents, and tests of different null hypothesis all in the same framework. Because our association tests were performed in the regions showing linkage evidence, our null hypothesis is linkage but no association. To account for the correlation among relatives caused by linkage, we used the empirical variance [13] in calculating test statistic. The results of FBAT analyses at each SNP were combined using Fisher's method as described above.

## Results

Four regions were found showing significant evidence for linkage (LOD > 3.6 [14]) in the meta-analysis of initial genome scans. The markers with highest LOD score at these regions are: D01S0023 (LOD = 7.7) on chromosome 1, D03S0127 (LOD = 19.0) on chromosome 3, D05S0173 (LOD = 13.9) on chromosome 5, and D09S0348 (LOD = 11.2) on chromosome 9. Significant evidence for linkage was also found with the same markers in the pooled data analysis: D01S0023 (LOD = 9.8), D03S0127 (LOD = 20.2), D05S0173 (LOD = 15.6), and D09S0348 (LOD = 11.6).

Fine mapping of KPD by linkage analysis was further performed at these regions by analyzing additional SNP markers. The results from meta-analysis and pooled data analysis are presented in Table 1. To further narrow down the regions containing the disease loci, SNPs within 1-LOD intervals were tested for association using FBAT. A total of 17, 26, 20, and 20 SNPs were tested at linkage regions on chromosomes 1, 3, 5, and 9. In the HWE tests, only one SNP, B01T0564, significantly deviated from the equilibrium (*p *= 0.0001) after adjustment for multiple testing in pooled data. LD was calculated around the linkage regions on chromosomes 1, 3, 5, and 9. The LD measurements from individual replicate and pooled data are similar. We defined strong LD as D' > 0.90 or r^2 ^> 0.30 [15]. No strong LD was found at the linkage region on chromosome 1 (maximum D' = 0.683 and maximum r^2 ^= 0.024). Strong LD was found around B03T3063-B03T3065 region on chromosome 3, B05T4141-B054143 region on chromosome 5, and B09T8337-B09T8339 region on chromosome 9.

The FBAT results for markers with nominal significance (*p *< 0.05) in either meta-analysis or pooled data analysis are presented in Table 2. Analyses using SNPSpD showed that the overall correlations across all SNPs in each region are weak and could be ignored. Thus multiple testing was adjusted using Bonferroni correction, which gave α = 0.05/83 = 0.0006 as the adjusted significance level. Significant association was detected for 8 SNPs (B03T3056, B03T3037, B03T3058, C03R0281 on chromosome 3; B05T4136 on chromosome 5; B09T8331, B09T8333 and B09T8340 on chromosome 9) in both analyses based on α = 0.0006. Two SNPs on chromosome 9 achieved significance only in pooled data analysis. They are C09R0765 (*p *= 0.0004) and B09T8341 (*p *< 0.0001).

No significant association was detected based on α = 0.0006 for chromosome 1 in either analysis. This could be caused by the small genetic effect, low frequency of causal disease mutation, or low LD between causal SNP with other SNP markers.

## Discussion

All 4 regions containing the major loci were correctly identified in the 2 combined linkage analyses, although no single replicate showed significant linkage evidence at all the regions. However, no significant linkage was found for the 2 modified loci on chromosome 2 and 10 even with combined analyses. This is most likely caused by the small effects of these 2 loci on the disease phenotypes. The linkage results are consistent for both analyses with the similar 1-LOD interval and significance level. However, the maximum LOD scores at the linkage regions are consistently lower in the meta-analysis than those in the pooled raw data analysis, which means that we may lose some power to detect linkage using meta-analysis although the loss is limited in our case.

We concentrated on single-SNP analysis in our association studies. The results of association analyses are quite consistent in both combined analyses. Significant association (*p *< 0.0006) was detected in both analyses for 8 out of 10 SNPs showing significant association in either analysis (Table 2). Two SNPs achieved significance only in pooled data analysis, which suggests that the power to detect association may be also higher in pooled data analysis.

An important assumption of meta-analysis using Fisher's method of combining *p*-values is that the primary studies are independent from each other. Bias could be caused by including studies with overlapping samples in the meta-analysis. This could be avoided by a careful check of authorship of the publications and detailed information about these studies. However, this may not be possible to do under some circumstances. If a serious overlap is found, an easy solution to prevent such bias is to leave some studies out. However, we may waste useful information by doing this. New statistical methods will be needed to incorporate such dependency in the meta-analysis.

The 10 replicates we chose for this analysis differ only in sampling and no heterogeneity exists among them. Our results show that meta-analysis has a similar power to detect linkage and association compared with pooled data analysis under homogeneous condition. In reality, different studies could differ in study design, marker set, statistical analysis and etc., further investigation need to be done to evaluate the effect of these factors on meta-analysis.

## Conclusion

Our analysis showed that, under homogeneous condition, the results from meta-analysis with Fisher's method and pooled data analysis are similar and the loss of power to detect linkage and association is limited for meta-analysis. Thus, meta-analysis can provide an overall evaluation of linkage and association when the original raw data is not available under this condition. More studies need to be done to investigate the power of meta-analysis when heterogeneity exists among primary studies.

## Abbreviations

GSMA: Genome-scan meta-analysis

HE: Haseman-Elston

HWE: Hardy-Weinberg equilibrium

IBD: Identical by descent

KPD: Kofendrerd Personality Disorder

LD: Linkage disequilibrium

SNP: Single-nucleotide polymorphism

## Figures and Tables

**Table 1:** Fine mapping results from meta-analysis and pooling raw data

		LOD		
			
Chromosome	Marker	Meta-analysis	Pooled raw data	1-LOD Region
1	C01R0052	11.6	13.2	D01S0023-C01R0053
3	C03R0281	19.0	20.9	C03R0279-C03R0281
5	C05R0380	14.6	16.9	C05R0379-C05R0381
9	C09R0765	14.3	15.0	C09R0764-C09R0766

**Table 2:** Result of family-based association test using FBAT

SNP^a^	*p*-meta	*p*-all
Chromosome 1		
B01T0545	0.0138	0.1447
B01T0549	0.0341	0.0293
B01T0551	0.1946	0.0398
B01T0558	0.3482	0.0487
C01R0052	0.0316	0.3425
		
Chromosome 3		
B03T3052	0.0329	0.0043
**B03T3056**	**<0.0001**	**<0.0001**
**B03T3057**	**<0.0001**	**<0.0001**
**B03T3058**	**<0.0001**	**<0.0001**
**C03R0281**	**<0.0001**	**<0.0001**
B03T3066	0.0584	0.0021
		
Chromosome 5		
**B05T4136**	**0.0006**	**<0.0001**
B05T4143	0.0021	0.0171
B05T4145	0.5868	0.0409
B05T4146	0.0016	0.0065
		
Chromosome 9		
**B09T8331**	**<0.0001**	**<0.0001**
**B09T8333**	**<0.0001**	**<0.0001**
C09R0765	0.0449	**0.0004**
B09T8337	0.1199	0.0010
B09T8338	0.5018	0.0439
**B09T8340**	**<0.0001**	**<0.0001**
B09T8341	0.0074	**<0.0001**
C09R0766	0.4118	0.0373

^a^SNPs are highlighted in bold if the *p*-values are less than 0.0006 in both combined analyses.
